# Special Issue “Cell Biology in Diabetes and Diabetic Complications”

**DOI:** 10.3390/ijms262412009

**Published:** 2025-12-13

**Authors:** Francesca Conserva, Paola Pontrelli

**Affiliations:** Department of Precision and Regenerative Medicine and Ionian Area (DIMEPRE-J), University of Bari Aldo Moro, Piazza Giulio Cesare n. 11, 70124 Bari, Italy

## 1. Introduction

Globally, diabetes mellitus represents a growing health challenge due to its metabolic dysregulation and the complex nature of its micro- and macrovascular complications such as diabetic kidney disease (DKD), diabetic retinopathy, cardiovascular disease and diabetic neuropathy [1,2,3]. 

As a result of recent pharmacological advances, glycemic control and clinical management have improved and many patients now live longer. Longer durations of disease, however, mean that new long-term diabetes-related complications continue to emerge, including subtle vascular and immune-mediated pathologies that may remain undetected using standard clinical markers [4].

Understanding the molecular changes induced by hyperglycaemia, oxidative stress, inflammation, and immune dysregulation therefore remains essential. Precise mechanistic insight is required to discover early biomarkers, develop targeted treatments, and ultimately shift therapeutic approaches from management toward true prevention.

**Figure 1:**
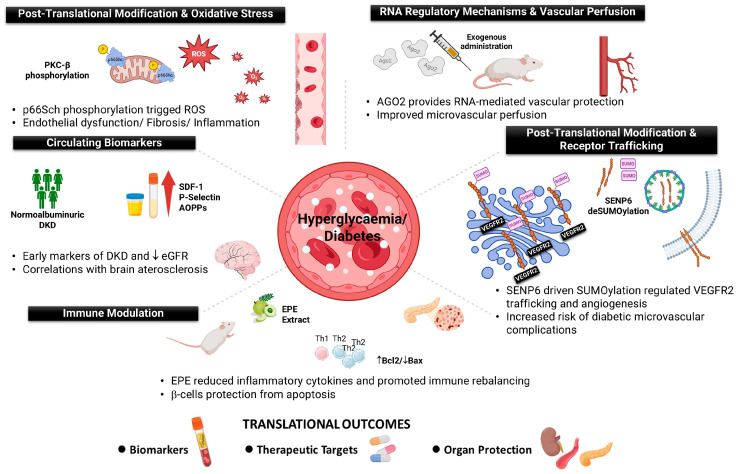
Molecular drivers of diabetic complications: redox stress, immune dysregulation, and repair pathways. The Post-Translational Modifications & Oxidative Stress panel: In response to high glucose, p66Shc undergoes PKC-β-dependent phosphorylation and translocates to mitochondria, where it promotes the generation of reactive oxygen species. This oxidative imbalance contributes directly to endothelial dysfunction, inflammation and fibrosis. The RNA Regulatory Mechanisms & Vascular Perfusion panel: The exogenous administration of Ago2 expression in the cavernous tissue of streptozotocin (STZ)-induced type-1 diabetic mice improves angiogenesis and ultimately restores erectile function. The Post-Translational Modifications & Receptor Trafficking panel: SUMOylated VEGFR2 is trapped in the Golgi, and SENP6-mediated deSUMOylation modulates the trafficking of VEGFR2 to the membrane, leading to microvascular remodeling. The Immune Modulation panel: The administration of *Phyllanthus emblica* Extract (EPE) promotes immunomodulation, conferring b-cell protection from apoptosis. The Circulation Biomarkers panel: In normoalbuminuric patients with diabetes, it is possible to detect early biomarkers of diabetic kidney disease (DKD) in serum and urine samples that are also correlated to cerebral atherosclerosis.

This Special Issue of the International Journal of Molecular Sciences, “Cell Biology in Diabetes and Diabetic Complications”, was designed to highlight research that advances this objective. The collected articles address molecular mechanisms including post-translational regulation, redox signaling, immune modulation, biomarker discovery, and RNA biology, each contributing toward earlier detection and more effective intervention. See Figure 1 for a graphical summary of all the molecular pathways described in this Special Issue.

## 2. Highlights of Specific Molecular Mechanisms That Promote Diabetic Complications Included in This Special Issue

### 2.1. Oxidative Stress and Redox Imbalance 

Oxidative stress is a defining molecular event across diabetic complications. Chronic hyperglycaemia drives the excessive production of reactive oxygen species (ROS), mitochondrial dysfunction, impaired antioxidant defenses, and damage to nucleic acids, proteins, and lipids [5]. This redox imbalance alters intracellular signaling, promotes endothelial dysfunction, contributes to extracellular matrix remodeling and fibrosis, and can trigger inflammatory cascades. In recent years, redox-sensitive regulatory proteins have gained attention as both mechanistic drivers and potential therapeutic targets, as exemplified by the works summarized below.

#### 2.1.1. Redox Protein p66Shc and Emerging Complications

Biondi et al. explore the role of p66Shc as a central redox regulator, showing its involvement across multiple diabetic complications including mitochondrial dysfunction, vascular injury, inflammation, and fibrosis [6]. p66Shc is a key redox-regulating protein whose expression and activation consistently increase under hyperglycemic conditions, where it functions as a molecular amplifier of oxidative stress and cellular injury [7]. In response to high levels of glucose, p66Shc undergoes PKC-β-dependent phosphorylation and translocates to mitochondria, where it promotes the generation of reactive oxygen species through the oxidation of cytochrome c, leading to mitochondrial dysfunction, impaired ATP production, and increased susceptibility to apoptosis [8]. This oxidative imbalance contributes directly to endothelial dysfunction, reduced nitric oxide bioavailability, vascular inflammation, and accelerated atherosclerosis. In the kidney, p66Shc drives podocyte loss, tubular epithelial cell injury, glomerulosclerosis, and interstitial fibrosis, and its genetic deletion protects mice from diabetic renal damage, confirming a causal role [9]. In parallel, p66Shc amplifies inflammatory signaling by enhancing NF-κB activity and cytokine production, creating a self-reinforcing loop between oxidative stress and inflammation [10]. Since p66Shc links metabolic stress to downstream cellular damage, it emerges both as a mechanistic node and as a potential therapeutic target. Modulating its activity could mitigate oxidative injury across multiple organs and tissues affected by diabetes.

#### 2.1.2. RNA-Binding Protein Ago2 as Vascular Repair Agent

Liu et al. examine the therapeutic potential of Argonaute 2 (Ago2)—the master component of the microRNA-induced silencing complex (miRNA-RISC) and regulator of RNA interference—in diabetic mice. The authors demonstrate that the expression of Ago2 is reduced under hyperglycaemia in the cavernous tissue of streptozotocin (STZ)-induced type 1 diabetic mice and that the restoration of its expression improves angiogenesis, reduces the accumulation of ROS, and promotes endothelial proliferation, ultimately restoring erectile function [11]. This work elegantly shows that RNA effector pathways can be therapeutically leveraged and that the modulation of RNA effector machinery can translate into organ-level functional benefits with implications that extend beyond erectile dysfunction.

### 2.2. Post-Translational Modifications and Protein Trafficking

Post-translational modifications (PTMs) such as phosphorylation, SUMOylation, acetylation, and ubiquitination play critical roles in regulating protein stability, localization, and signaling [12,13,14,15]. In diabetes, altered PTMs can drive cellular fate decisions, modify receptor sensitivity, or disrupt intracellular trafficking pathways [16]. Recent discoveries highlight that protein function is not uniquely determined by expression levels but by subcellular localization and dynamic modification states, which are increasingly being recognized as therapeutic intervention points [17].

#### Post-Translational Regulation of Angiogenic Signaling

He et al. describe how advanced glycation end products (AGEs) lead to the upregulation of VEGFR2 via Nrf2 and increase the expression of SUMO-specific protease 6 (SENP6) deSUMOylating enzyme. The deSUMOylation of VEGFR2 reduces its accumulation in the Golgi and promotes its transportation to the endothelial cell membrane, increasing angiogenesis [18]. The SENP6-VEGFR2 interaction network shows how post-translational modification and intracellular trafficking, rather than expression alone, affect endothelial behavior and disease progression.

### 2.3. Biomarker Discovery

The identification of circulating biomarkers that reflect early microvascular stress, or immune dysregulation in the context of diabetic kidney disease (DKD), is increasingly being pursued by both academia and pharmaceutical industries, since classical markers such as albuminuria may fail to detect early injury. Recent advances in proteomics, metabolomics, extracellular vesicle profiling, and immune phenotyping indicate the need for a more diverse biomarker landscape, including chemokines, adhesion molecules, post-translational modification byproducts, mitochondrial stress signatures, and cytokine profiles [19].

#### 2.3.1. Biomarkers of Early Vascular Dysfunction in DKD

Petrica et al. study human patients with type 2 diabetes and normoalbuminuric diabetic kidney disease (DKD), finding that elevated stromal cell-derived factor-1 (SDF-1), P-selectin, and advanced oxidation protein products (AOPPs), together with evidence of mitochondrial dysfunction, correlate with early cerebral vascular changes [20]. As these markers present before standard renal indicators manifest, they may serve for earlier risk stratification and intervention.

#### 2.3.2. Immunoregulatory and Antidiabetic Effects of *Phyllanthus emblica* L. Extract

Lin et al. investigate an ethyl acetate extract from *Phyllanthus emblica* L. (EPE) in non-obese diabetic (NOD) mice (both in spontaneous and in cyclophosphamide-accelerated models). The authors describe the beneficial properties of EPE in terms of lowering blood glucose and HbA1c, increasing insulin levels, modulating the immune responses (decreasing Th1/Th17 proinflammatory cytokines and increasing Th2/Treg cytokines), and improving β-cell survival [21]. This work suggests both a biomarker-rich profile (cytokines and cell subset changes) and the therapeutic potential of the natural extract in regulating immune dysregulation in type 1 diabetes.

## 3. Final Considerations and Future Directions

Several shared themes emerge from these studies. First, oxidative stress and redox imbalance remain central to the pathogenesis of diabetic complications, with p66Shc, AOPPs, and Ago2 all highlighting different aspects of this process. Second, post-translational modifications and protein trafficking, such as the SUMOylation of VEGFR2 or Ago2-mediated regulation of ROS, are increasingly being recognized as crucial determinants of cellular fate. Together, these works highlight the need to integrate molecular signaling, protein and cellular localization, and intracellular communication.

The future of diabetes research will benefit from integration; advances in omics technologies, particularly single-cell transcriptomics, proteomics, and spatial imaging, will allow us to explore cellular heterogeneity and molecular states at a previously unimaginable resolution. Biomarker discovery remains a priority, particularly for markers that can predict complications before irreversible injury. At the same time, mechanistic insights into regulators such as p66Shc, SENP6, and Ago2 raise the possibility of targeted interventions or drug repurposing. Importantly, translational progress will require multidisciplinary collaboration between molecular and cellular biologists, clinical investigators and pharmacologists.

The contributions to this Special Issue demonstrate how detailed cell biology can inform translational medicine in diabetes. From redox proteins to receptor trafficking and adipose secretomes to circulating biomarkers, these studies not only illustrate the heterogeneous mechanistic landscape that underlies diabetic complications, but they also point to actionable targets and measurable signals that may one day change patient outcomes. We are grateful to the authors for their excellent contributions and invite readers to engage with these works as we continue our collective efforts to understand and mitigate the molecular drivers of diabetic disease.
